# A Call for Action: Lessons Learned From a Pilot to Share a Complex, Linked COVID-19 Cohort Dataset for Open Science

**DOI:** 10.2196/63996

**Published:** 2025-02-11

**Authors:** Clara Amid, Martine Y van Roode, Gabriele Rinck, Janko van Beek, Rory D de Vries, Gijsbert P van Nierop, Eric C M van Gorp, Frank Tobian, Bas B Oude Munnink, Reina S Sikkema, Thomas Jaenisch, Guy Cochrane, Marion P G Koopmans

**Affiliations:** 1 Department of Viroscience, Erasmus Medical Center (Erasmus MC), Dr Molewaterplein 40, Rotterdam, 3015 GD, Netherlands, 31 107044770; 2 European Molecular Biology Laboratory (EMBL), European Bioinformatics Institute (EBI), Wellcome Genome Campus, Hinxton, Cambridgeshire, United Kingdom; 3 Heidelberg Institute of Global Health (HIGH), Heidelberg University Hospital (UKHD), Heidelberg, Germany; 4 Center for Global Health, Colorado School of Public Health, Aurora, CO, United States; 5 Pandemic and Disaster Preparedness Center (PDPC), Rotterdam, Netherlands

**Keywords:** data sharing, data management, open science, COVID-19, emerging infectious disease, global health

## Abstract

The COVID-19 pandemic proved how sharing of genomic sequences in a timely manner, as well as early detection and surveillance of variants and characterization of their clinical impacts, helped to inform public health responses. However, the area of (re)emerging infectious diseases and our global connectivity require interdisciplinary collaborations to happen at local, national and international levels and connecting data to understand the linkages between all factors involved. Here, we describe experiences and lessons learned from a COVID-19 pilot study aimed at developing a model for storage and sharing linked laboratory data and clinical-epidemiological data using European open science infrastructure. We provide insights into the barriers and complexities of internationally sharing linked, complex cohort datasets from opportunistic studies for connected data analyses. An analytical timeline of events, describing key actions and delays in the execution of the pilot, and a critical path, defining steps in the process of internationally sharing a linked cohort dataset are included. The pilot showed how building on existing infrastructure that had previously been developed within the European Nucleotide Archive at the European Molecular Biology Laboratory-European Bioinformatics Institute for pathogen genomics data sharing, allowed the rapid development of connected “data hubs.” These data hubs were required to link human clinical-epidemiological data under controlled access with open high dimensional laboratory data, under FAIR (Findable, Accessible, Interoperable, Reusable) principles. Based on our own experiences, we call for action and make recommendations to support and to improve data sharing for outbreak preparedness and response.

## Introduction

The COVID-19 pandemic proved in a real-case scenario how rapid FAIR (Findable, Accessible, Interoperable, Reusable) [1] sequence data sharing was used to inform public health response policies [2]. An international collaboration, the Reconciliation of Cohort data in Infectious Diseases (ReCoDID) [3], a 4-year project that began in January 2019, was funded by the European Commission’s Horizon 2020 programme with a focus on the global response to emerging pathogens. Building on existing infrastructures and partnerships, the consortium was funded to develop a sustainable model for the storage, curation, and analyses of complex datasets collected from infectious disease related cohorts to facilitate and speed up outbreak research. In 2020, a pilot study was developed within this project using COVID-19 data from Erasmus Medical Center (Erasmus MC) as a use-case. The incentive was that in an ongoing global COVID-19 pandemic, linking data for connected data analysis with the potential to better understand the disease would be of public interest and of public health importance. The aim of the pilot was to test the potential for sharing linked laboratory data and clinical-epidemiological (CE) data in the evolving European infrastructure for open science in a centralized model. The centralized model, as opposed to a decentralized or federated data sharing model, was chosen for this pilot because we wanted to build on existing infrastructure [4,5]. The model has the potential of hosting and curation of large datasets, the benefit of standardized data input and output leading to interoperability, easing re-analysis by others necessary for improved prediction and preparedness on global, EU and national level, as was needed for a newly emerged outbreak. A decentralized or federated data sharing model has the benefit of not needing to move the data, but the consequences often manifest themselves in no sharing or limited sharing and linking of datasets, difficulty to apply federated data analyses across a wide range of data formats and sources, with predictions and preparedness happening more at local or regional level. Given the diversity of datasets and the complexity of sharing data from clinical research and in line with the rapidly evolving situation during the COVID-19 pandemic, we wanted to learn what it would take to release linked data from opportunistic studies, rather than pre-approved cohort data.

Here, we describe experiences and lessons learned from this pilot, providing insights into the complexities of internationally sharing linked, cohort datasets for connected data analyses.

## Pilot Set Up

The following sections have references to the timeline (Multimedia Appendix 1) in which the steps taken to set up and complete a pilot with a small but complex, linked dataset from opportunistic COVID-19 clinical and laboratory research studies as done in (academic) hospital settings are shown. We divided these steps into the following categories: data identification and acquisition, ethics and legal requirements (data protection), data harmonization, and data storage and delivery. Starting in November 2020, meetings took place with basic researchers and clinical scientists who had generated COVID-19 data as part of pandemic related research [6,7]. The drafted data map illustrated how complex the seemingly “simple” datasets (clinical, genomic, pathogen genomic, and high-dimensional laboratory) were. Different data sharing rules and governance for different parts of the datasets existed, and different identifiers and platforms for data capture and storage had been used. It became apparent that these procedures were not unique to COVID-19 pandemic and were common practice at the hospital. Finally, after a further meeting with the involved scientists to discuss datasets that could be linked in a pilot, a small cohort of 151 patients (intensive care unit and non–intensive care unit) who had tested SARS-CoV-2 positive (reverse transcription polymerase chain reaction) between May 2020 and May 2021 was selected for inclusion in the pilot, for whom several data types were partially available, including SARS-CoV-2 viral whole genomes, protein-microarray serological readouts, T- and B-cell data as well as CE data (data identification and acquisition).

**Figure 1.**
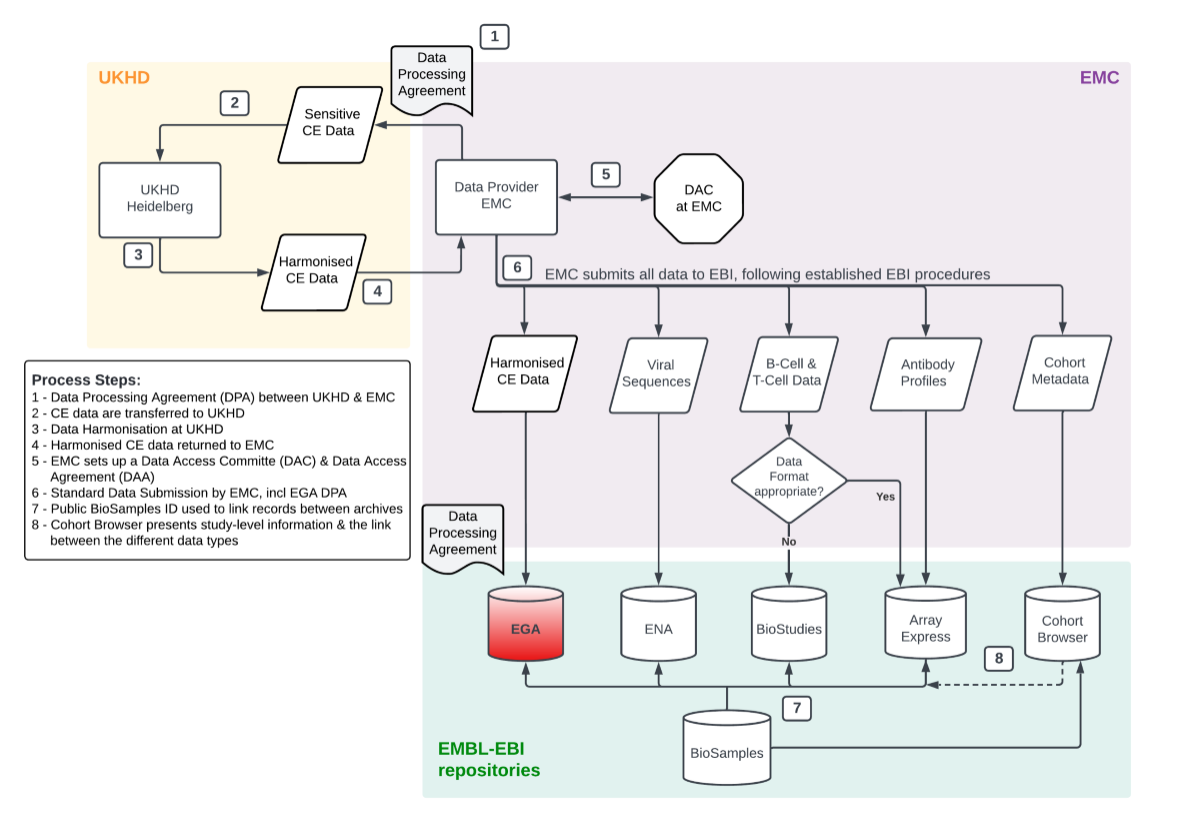
Overview of Erasmus Medical Center (EMC)’s pilot dataflow, linkage and presentation for EMC’s connected dataset pilot. The roles of the participating institutes within this pilot were as follows. EMC: mobilization and consolidation of the datasets (data identification and acquisition) by the EMC team at the source within EMC, participant-level and derived BioSample record registration to include metadata about biological samples, linking and submission of all data to repositories within the European Molecular Biology Laboratory-European Bioinformatics Institute (EMBL-EBI) infrastructure including deposition of clinical-epidemiological (CE) data under controlled access at European Genome-Phenome (EGA), depositions of viral sequences at the European Nucleotide Archive (ENA), B-cell and T-cell data, as well as antibody profiles at BioStudies and ArrayExpress repositories, respectively, and setting up a data access committee (DAC) and data access agreement (DAA) (data storage and delivery); University Hospital Heidelberg (UKHD): harmonization of the CE data and data dictionary at UKHD according to international standards (data harmonization); EMBL-EBI: provided the process for data linking on a participant level, including linking of the CE data to omics data types, using the hierarchical BioSamples database model and presentation of EMC’s cohort study in the Reconciliation of Cohort data in Infectious Diseases Cohort Browser (data storage and delivery). All steps taken in this process (numbered arrows) are also numbered in the key entitled “Process Steps.”

Due to the extensive legal discussions around data sharing (ethics and legal requirements), the implementation of the long-term model of the ReCoDID data workflow was brought to a standstill (see “Enablers and Barriers Affecting the Execution of the Pilot” ). Therefore, a slightly deviated dataflow model for the pilot was agreed on. The deviation was solely focused on CE data and meant that instead of Heidelberg University Hospital (UKHD) transferring harmonized CE data directly to the European Genome-Phenome Archive (European Molecular Biology Laboratory-European Bioinformatics Institute [EMBL-EBI]), the harmonized CE data were transferred back from UKHD to Erasmus MC, and Erasmus MC carried out the CE data submission to the European Genome-Phenome Archive, including a further data processing agreement (Figure 1). This change was necessary to circumvent one of the barriers (”Adherence to GDPR, and differences in its interpretation” in Table 1) and to move forward with the pilot and to facilitate the development of the connected datasets platform. Furthermore, the Erasmus MC’s legal and privacy offices and the institute’s COVID-19 clinical database team (established in June 2020) were contacted to discuss the aim of the pilot, the access procedures to the CE data, as well as all other steps and legal requirements for sharing the CE data internationally. For this purpose, a Data Protection Impact Assessment was carried out. A data access request for CE data though the Erasmus MC’s COVID-19 clinical database was also required (ethics and legal requirements).

**Table 1.** Enablers and barriers that affected the Erasmus Medical Center (Erasmus MC) pilot’s execution.

Description of enablers and barriers	Stakeholders	Category of actions
Enabler Description		
Active support from legal advisors or GDPR^a^ specialists. Navigating the complexity of regulations governing data sharing, such as the GDPR, and to apply these accordingly, requires expert privacy and legal knowledge. The active support from legal or GDPR specialists was therefore pivotal in defining the process of dataflow within ReCoDID^b^.	Academic hospitals or institutes	Ethics and legal requirements
Building forward on existing infrastructure for sharing (biomedical) data, allowed the rapid development of the connected data hubs to include controlled access sensitive data for/in this pilot.	Researchers; European Union and national funding agencies	Data storage and delivery
Building forward on existing collaborations, as developed in ReCoDID and COMPARE (Collaborative Management Platform for Detection and Analyses of (Re)emerging and Foodborne Outbreaks in Europe) [8], facilitated the steps in the pilot and helped to further build trust necessary for data sharing between participants. The collaboration between the data provider or controller (Erasmus MC) and the respective EMBL-EBI^c^ repositories, as well as between the EMBL-EBI repositories, was needed in this pilot to drive further developments and to extend the existing infrastructures to host and present connected datasets.	Researchers; European Union and national funding agencies	All
Dedicated data scientist championing the pilot. Acquiring data from different sources and systems and collected by various specialist teams, linking such data, and ensuring the (legal) requirements are fulfilled before sharing such data, is cumbersome and time-consuming. A dedicated data scientist, supported by encouragement and advocacy of the principal investigator for data sharing amongst researchers, ensured the pilot kept moving ahead.	Academic hospitals or institutes	All
Barrier Description		
Adhering to GDPR, and differences in its interpretation. The data workflow gave rise to legal issues in the context of the GDPR, with regards to GDPR roles (eg, controller vs processor), potential liabilities, different interpretations of Article 49 (d) (Derogations for specific situations, important reasons of public interest), also in regard to the transfer of data to international organizations (here: EMBL-EBI), and resulted in long-lasting legal discussions to define the process of dataflow within ReCoDID.	European Union and national governments; supervisory authorities^d^	Ethics and legal requirements
Insufficient FAIRification^e^ and sharing of initial data. Lack of both mandatory FAIRification and sharing rules and specialized staff in inter-pandemic times led to a situation during the pandemic, when time and resources were naturally scarce, often differently prioritized and competing priorities existed, where FAIRification and data sharing was not the main priority. This also led to a situation where harmonization of CE^f^ data according to international standards (WHO-ISARIC^g^ format) [9] was delayed or not completed.	Academic hospitals or institutes; researchers; European Union and national funding agencies	Data identification and acquisition; data storage and delivery
Insufficient interoperability of the data. Establishing linkages between CE data and corresponding high-dimensional lab data was challenging, and the availability of overlapping data types was limited. Various identifiers were used for CE data and derived high-dimensional laboratory data, and metadata was not fully harmonized, making it difficult to establish linkages. Furthermore, as high-dimensional data types were generated in separately funded projects, a limited amount of overlap in high-dimensional data types existed for the patients.	Academic hospitals or institutes; researchers; European Union and national funding agencies	Data identification and acquisition
Issues contributing to difficulty in CE data acquisition		
Competing priorities: there was a plethora of clinical studies to be entered into the institute’s COVID-19 clinical database. Consequently, there were difficulties in getting approval for prioritization of data entry and data access for this project. Finally, over time there was a reduced interest with decreasing burden of COVID-19 in completing the data entry of CE data. Time consuming and resource intensive handling and storage of CE data: CE data was stored at different specialized hospital health record systems, that allowed only partial automation of data entry into the institute’s COVID-19 clinical database, and records needed to be administered individually. During a time when clinical services were overburdened with COVID-19 cases this resulted in a backlog of CE data that needed to be entered into the institute’s database. Discontinued institutional funding and support for its COVID-19 clinical database: after one year (in 2021), the database was discontinued and subsequently placed under different management. This delay also meant that additional requirements, eg, related to data protection and ethics, had to be met before CE data could be accessed and shared.	Academic hospitals or institutes	Data identification and acquisition

a GDPR: General Data Protection Regulation.

b ReCoDID: Reconciliation of Cohort data in Infectious Diseases.

c EMBL-EBI: European Molecular Biology Laboratory-European Bioinformatics Institute.

d The supervisory authorities (barrier “Adhering to GDPR, and difference in its interpretation”) refer to the independent public authorities in each Member State responsible for monitoring the application of the GDPR.

e FAIR: Findable, Accessible, Interoperable, Reusable.

f CE: clinical-epidemiological.

g WHO-ISARIC: World Health Organization-International Severe Acute Respiratory and emerging Infection Consortium.

## Linking of Different Data Types

At the time of conducting this pilot, submission of SARS-CoV-2 viral genomes to repositories such as EMBL-EBI’s European Nucleotide Archive (for raw sequences) and GISAID [10] (for consensus sequences) was done routinely, other laboratory datasets (protein-microarrays, T- and B-cell data) were not shared. To link SARS-CoV-2 genomes to the other datasets a check was done to verify whether viral genomes had been submitted from the 151 individuals. This exercise required iterative rounds of mapping 4 different identifiers. The internal mapping exercise further revealed the partiality of all other datasets for the individuals included in this cohort. Serum antibody profiles generated using protein-microarrays were available for 40 patients, T-cell phenotype and reactivity data for 28 patients and clonal antibody cross-reactivity data using B-cell profiling for 17 patients out of 151 selected patients in the cohort. For 7 patients out of 151 all data types were available.

## Configuration of Data Infrastructure and Data Delivery

With the exception of the viral genomes that were routinely submitted to known repositories (refer to “Linking of different data types” section), for storage and linkage of the dataset it was needed to identify suitable respositories for all other laboratory datasets. This meant establishing where new pilot data types could be hosted, and how the EMBL-EBI system could be leveraged to link all pilot datasets within the EMBL-EBI system, so that the datasets could be eventually also “presented” through a Cohort browser [11]. Although the EMBL-EBI repositories were not fully designed to host one of our data types (T-cell) it was possible to submit the outputs with some adjustments to the format as proof of concept. This leaves room for discussion between the respective researchers and the EMBL-EBI to allow for a more routine application.

The upload of the last Erasmus MC pilot datasets was completed in November 2022. To link all datasets EMBL-EBI’s BioSample database [12] was used with a hierarchical model allowing linkage between individuals and their derived samples. With the completion of linking all data types on participant level and presentation of all datasets through the Cohort browser, the pilot was considered as completed (data storage and delivery) in December 2022 [13]. Study level metadata and links to the datasets in the corresponding repositories can be accessed through the Cohort browser and the BioSamples browser at EMBL-EBI [14].

## Enablers and Barriers Affecting the Execution of the Pilot

Several enablers and barriers were identified that either facilitated or hampered the execution of this pilot, respectively (Table 1). The main category of actions affected by these barriers and enablers was also identified, as well as the main stakeholder(s) to influence the enablers and barriers.

Several barriers significantly delayed the sharing of a linked dataset in this pilot. The barriers associated with complying with ethics and legal requirements, and with data identification and acquisition, caused the most evidential delays. Some of these barriers tended to reinforce each other, particularly due to shared underlying issues, such as the siloed nature of clinical and complex laboratory data collection and storage. At the start of the pilot, CE data was in different hospital systems, and not yet integrated into the (newly established) institute’s database for COVID-19 patient related CE data (”Issues contributing to difficulty in CE data acquisition” in Table 1). Derived high dimensional laboratory data was stored in different systems, separate from patient and diagnostic databases (“Insufficient FAIRification and sharing of initial data”). This was a result of data being gathered by multiple different specialist teams, often for separately funded projects, as well as the lack of linked data infrastructures capturing both patient and (raw) laboratory data, which is typical for hospital systems. Furthermore, the COVID-19 pandemic was characterized by a fragmented funding landscape. Many projects were funded, often focusing on a specific research question or field, thereby reinforcing the siloed nature of specialist teams’ work. In addition, for each of these projects, patient sample materials were needed (eg, swabs, blood, and serum). Consequently, high-dimensional lab data were generated for only a subset of patients, based on availability of sample material and requirements of the specific research topic (”Insufficient interoperability of the data”).

Interestingly, the newly established institute’s COVID-19 clinical database could have in principle worked as an enabler for data sharing. It was designed with the aim for CE data to be collected, stored and retrieved from one central database, and potentially address the issue of CE data being siloed in different existing hospital IT systems and databases and improve the FAIR aspects of such data. Yet, a variety of issues, described in Table 1, caused significant delays in CE data acquisition and sharing.

Several factors contributed to or facilitated the sharing of a linked dataset in this pilot, which showed the willingness of the involved teams to overcome the barriers in the sharing of a complex, linked dataset.

## Critical Path for Sharing a Complex, Linked Dataset

**Figure 2.**
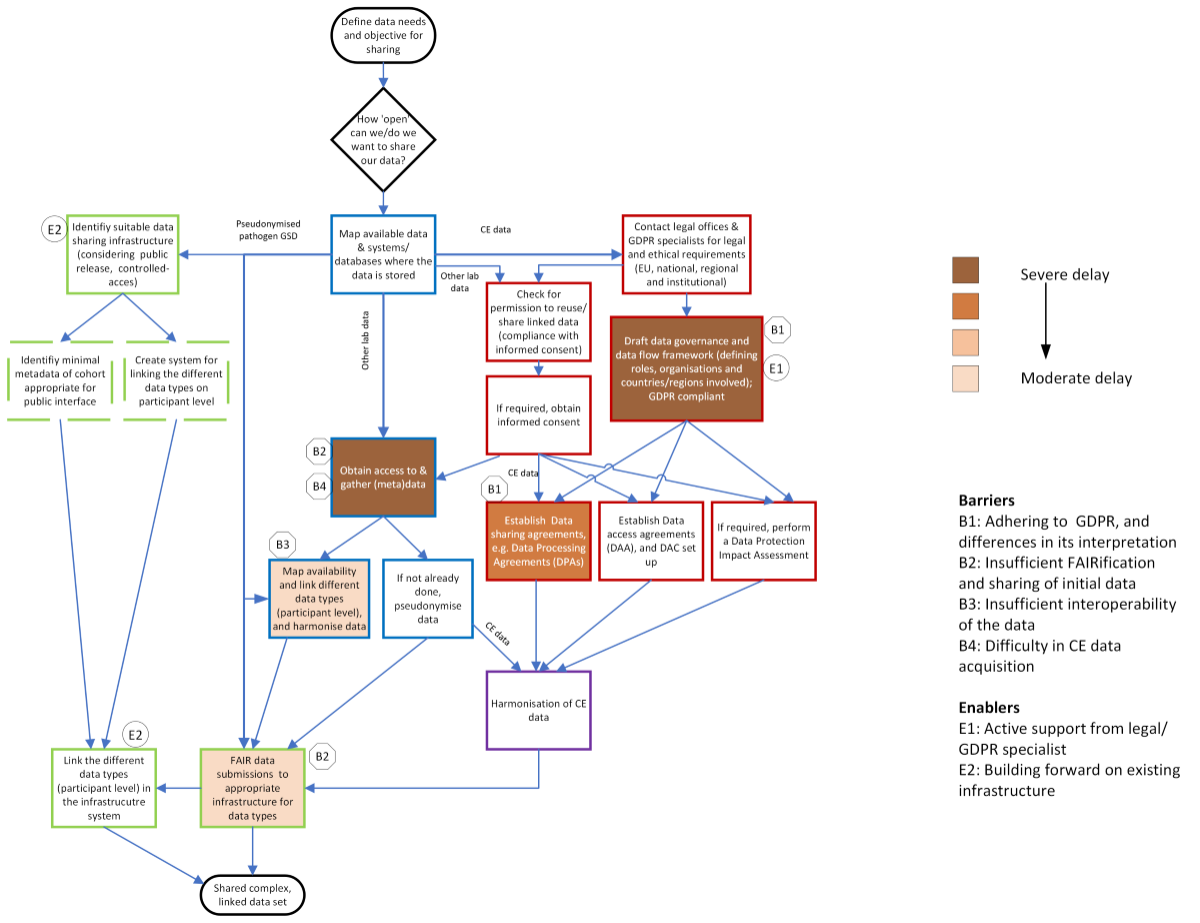
Critical path for sharing a complex, linked dataset. Two rounded boxes mark the start and endpoint; the diamond box represents a decision. Steps were classified into 4 categories of actions (color coding corresponds to timeline): data identification and acquisition (4 steps, shown in text boxes with blue margins), ethics and legal requirements (7 steps, shown in red), external data harmonization (1 step, shown in purple), and data storage and delivery (5 steps, shown in green). Dashed green boxes represent steps that were needed for this first pilot to build the system (European Molecular Biology Laboratory-European Bioinformatics Institute). Different types of data required different steps in this process, as shown for pseudonymized pathogen genetic sequence data (GSD), other laboratory datasets (other lab data), and clinical-epidemiological (CE) data. The identified barriers and enablers are indicated at the specific steps in the process that were mainly affected for our pilot; 2 enablers are not shown as these did not affect specific steps but acted as general facilitators of this process for our pilot. DAC: data access committee; EU: European Union; FAIR: Findable, Accessible, Interoperable, Reusable; GDPR: General Data Protection Regulation;

The critical path analysis (Figure 2) defined the steps in the process of sharing a complex, linked cohort dataset, showing the necessary order of steps or tasks to complete the submission and sharing of such datasets through an appropriate infrastructure, based on our pilot experiences. These steps were classified into the same 4 categories of actions introduced above, data identification and acquisition (4 steps), ethics and legal requirements (7 steps), external data harmonization (1 step), and data storage and delivery (5 steps). Most of the steps in this process could be worked on concurrently, depending on the availability of resources (eg, time of legal staff; availability of appropriate, and standardized templates). As shown in Figure 2, pathogen genetic sequence data (GSD) required a minimum number of steps (1 step), followed by other laboratory data (6 steps), whereas CE data required at least 10 steps. This also corresponds to the time for completion of all steps for each data type in our pilot, with the least amount of time for pathogen GSD. Figure 2 also shows how the identified barriers and enablers affected the process for this pilot.

## Mission (Im)possible: A Call for Action

The pilot demonstrated that sharing a linked cohort dataset using a centralized approach can succeed, but several crucial barriers (as described in Table 1) exist that cause long delays. Although these barriers were not unique to COVID-19 pandemic and have been extensively described previously [15-17], they persist today, as currently the burden to overcome or circumvent such barriers is placed on the individual researchers, projects, or institutes. However, the root causes of these barriers are systemic in nature. To alleviate these barriers, stakeholders at institutional, national and international or EU level (eg, governments, health care, research, journals, funders, and legal) need to work jointly toward building a model that not only supports but motivates data exchange by simplifying steps in the process of data sharing, taking privacy and legal aspects into account, and to reward researchers in new ways [18]. Below we describe some immediate actions to achieve this based on our experiences.

### Support With Privacy Questions and General Data Protection Regulation

We recommend creating a roadmap amongst EU countries for how to deal with common issues, to avoid (recurrent) lengthy legal discussions. Harmonization of regional and national implementation of GDPR, and alleviation of the disproportionate burden in legal compliance activities for data sharing with international organizations, needs to be achieved [19]. Finally, additional support should be provided by the European Union to their funded projects, for example, by providing legal guidance or a top up for projects to hire legal experts.

### Build and maintain FAIR Data Sharing During “Interpandemics”

Mandatory FAIRification of data in funded projects with funding for personnel to build and maintain FAIR data collections and FAIR sharing, and monitoring of and consequences for noncompliance. At the same time, more data repositories submitting to evaluation-based initiatives and accreditation processes [20-22], could help with a cultural change toward more FAIR data sharing. Furthermore, FAIRification and sharing of data should become standard practice during the education of bachelor, master, and PhD students.

### Decrease Fragmented Funding Landscape

Consideration for funding interdisciplinary projects to enable combined data sharing and, continued funding support for existing data infrastructures is key to sustaining the efforts of data FAIRification and sharing, both nationally and internationally. One clear example is the COVID-19 open data portal [23], an initiative launched at the explicit wish from the European Commission. It provides access to open data from a range of areas of expertise for use across sciences (biomedical and social sciences). A second example is the newly launched World Health Organization (WHO) pandemic hub, to develop “collaborative surveillance,” a new area that seeks to bring together data collected by different areas of expertise. This includes the classical epidemiological data that are the core of public health surveillance, but also data on climate parameters, and data on factors that influence disease dynamics.

### Willingness and Commitment Building (Culture Change)

We call for establishment of a common policy about data sharing allowing culture change at the EU or international level and for provision of resources to do so. One way of supporting culture change can be achieved by reflecting how researchers are recognized, rewarded, or assessed beyond publishing results in a journal. On short term, acknowledging data providers when reusing their datasets, and the use of citable data Digital Object Identifiers (DOI) pointing to their data could help (eg, as used for one of the datasets used in this pilot [24]). At institutional level, common policies about data sharing should also be established, and resources provided to implement these, to improve existing sharing norms and cultures.

### Standardization and Interoperability

Institutes need to prioritize standardization or reconciliation of existing databases and systems to tackle data siloes. Overarching infrastructures at University Hospitals that would allow and enhance standardization, harmonization, and linking of different data types locally and nationally could also increase interoperability at EU and international level [25].

## Discussion

Without action, barriers will persist, especially for sharing CE data, which hamper FAIR data sharing for infectious disease preparedness and response. The actions described here also contribute toward the commitment from closed to open research information, as put forward in the recent Barcelona Declaration [26]. The first and uppermost question needs to be answered at institutional and national level: Do people want to share data and what are they prepared to invest in terms of a collaborative effort to do so, if there was funding, GDPR and other support, for data sharing to move away from a felt “burden” to being a seen “benefit.” On the path toward more open data sharing researchers’ and other stakeholders’ concerns about sharing “their” data need to be addressed [15,27,28].

Meanwhile, decentralized or federated approaches are often preferred, as these circumvent barriers [16,29,30] and are less resource- and time intensive for (clinical) researchers and institutes. Federated networks, where decentralized but interconnected nodes allow data to be queried or analyzed by other nodes in the network without the data leaving its location, have been proposed as a solution and are being worked on to address the siloing of health data and current barriers to data sharing [31]. However, common obstacles to broader uptake of federated networks include the absence of data standards or limited adherence to existing standards, the complexity of designing, implementing and deploying federated solutions that preserve privacy [32]. The challenges must be addressed before federated networks can be implemented more widely, especially across national borders [31], but the question remains if full federation will ever be possible. Other concerns with federated approaches, such as the forthcoming European Health Data Space [33], are about significant delays in the authorization by national health data access bodies for reuse or secondary use of health data [34], and introduction of paywalls [35], potentially leading to inequity.

There is a need to streamline the timeliness of steps for sharing CE and other data, and to ensure no additional barriers are created in sharing a linked cohort dataset. Ongoing discussions to include digital sequence information, including pathogen GSD, under the Nagoya protocol [36], or the WHO CA+ [37], could potentially result in new barriers due to additional steps to complete legal procedures before pathogen GSD could be shared. This may considerably delay the prompt sharing and access to pathogen GSD, which has proven crucial in early detection, diagnostics, and identifying variants during the COVID-19 pandemic [2], while its impact on improving access and benefit sharing mechanisms remains uncertain [38].

The multidisciplinary nature of infectious disease research and collaborations reflects the complexity of research and public health questions which need to be understood and answered around (re-) emerging diseases. While certain answers can be found in isolation within a specific area of research and small numbers of patients, larger complex questions require a joint outlook and a combination of data sources to be examined together [39].

## Conclusions

Here we have shown the complexities in sharing a small but complex, linked dataset from opportunistic COVID-19 clinical and laboratory research studies as done in (academic) hospital settings. We also provided actionable elements to shorten the timeline to go through this process. Actions that are taken now to improve data sharing for outbreak preparedness and response will improve our ability to detect and respond to emerging threats in the future. We call upon governments, funders, global and regional organizations, scientists and their institutes, journals and industries to tackle known barriers hampering data sharing for infectious disease preparedness and response.

## Supplementary material

10.2196/63996 Multimedia Appendix 1 Supplementary material - timeline.

## Abbreviations

CE: clinical-epidemiological
EMBL-EBI: European Molecular Biology Laboratory-European Bioinformatics Institute
Erasmus MC: Erasmus Medical Center
FAIR: Findable, Accessible, Interoperable, Reusable
GDPR: General Data Protection Regulation
GSD: genetic sequence data
ReCoDID: Reconciliation of Cohort data in Infectious Diseases
UKHD: Universitätsklinikum Heidelberg (University Hospital Heidelberg)
WHO: World Health Organization

## References

[1] Wilkinson MD, Dumontier M, Aalbersberg IJJ (2016). The FAIR Guiding Principles for scientific data management and stewardship. Sci Data.

[2] Oude Munnink BB, Nieuwenhuijse DF, Stein M (2020). Rapid SARS-CoV-2 whole-genome sequencing and analysis for informed public health decision-making in the Netherlands. N Med.

[3] Reconciliation of Cohort data in infectious diseases. ReCoDID.

[4] Amid C, Pakseresht N, Silvester N (2019). The COMPARE data hubs. Database (Oxford).

[5] Rahman N, O’Cathail C, Zyoud A, Sokolov A, Oude Munnink B, Gruning B (2024). Mobilisation and analyses of publicly available SARS-CoV-2 data for pandemic responses. Microb Genom.

[6] Aguilar-Bretones M, Westerhuis BM, Raadsen MP (2021). Seasonal coronavirus-specific B cells with limited SARS-CoV-2 cross-reactivity dominate the IgG response in severe COVID-19. J Clin Invest.

[7] Lu L, Sikkema RS, Velkers FC (2021). Adaptation, spread and transmission of SARS-CoV-2 in farmed minks and associated humans in the Netherlands. Nat Commun.

[8] COMPARE Collaborative management platform for detection and analyses for (re-)emerging and foodborne outbreaks in Europe.

[9] International severe acute respiratory and emerging infection consortium. ISARIC.

[10] GISAID.

[11] Cohort Browser Infectious disease cohort studies shared at EMBL-EBI.

[12] Courtot M, Gupta D, Liyanage I, Xu F, Burdett T (2022). BioSamples database: FAIRer samples metadata to accelerate research data management. Nucleic Acids Res.

[13] Stroe O (2022). First linked dataset on Pathogens Portal: EMBL-EBI.

[14] BioSamples, EMBL-EBI Search results for ReCoDID COVID-19 pilot study.

[15] Ribeiro CDS, Roode MY, Haringhuizen GB, Koopmans MP, Claassen E, Burgwal LHM (2018). How ownership rights over microorganisms affect infectious disease control and innovation: A root-cause analysis of barriers to data sharing as experienced by key stakeholders. PLoS ONE.

[16] Bernier A, Molnár-Gábor F, Knoppers BM (2024). Reconciling the biomedical data commons and the GDPR: three lessons from the EUCAN ELSI collaboratory. Eur J Hum Genet.

[17] Vlahou A, Hallinan D, Apweiler R (2021). Data sharing under the general data protection regulation: time to harmonize law and research ethics?. Hypertension.

[18] Maxwell L, European Commission, Directorate-General for Research and Innovation (2022). Maximising investments in health research – FAIR data for a coordinated COVID-19 response– Workshop report: Publications Office of the European Union.

[19] (2024). Science europe feedback on the implementation of the GDPR. Science Europe.

[20] Lin D, Crabtree J, Dillo I (2020). The TRUST Principles for digital repositories. Sci Data.

[21] ELIXIR core data resources: ELIXIR Europe. ELIXIR.

[22] Resources, GCB Global Core Biodata Resources: Global Core Biodata Resources.

[23] COVID-19 Data Portal - accelerating scientific research through data.

[24] Oude Munnink B. ND, Sikkema R, Schapendonk C On behalf of the Dutch national COVID-19 response team SARS-CoV-2 whole-genome sequencing for informed public health decision making and outbreak tracking and tracing in the Netherlands.

[25] Rinaldi E, Stellmach C, Rajkumar NMR (2022). Harmonization and standardization of data for a pan-European cohort on SARS- CoV-2 pandemic. NPJ Digit Med.

[26] (2024). Barcelona declaration on open research information. https://barcelona-declaration.org/.

[27] Federer LM, Lu YL, Joubert DJ, Welsh J, Brandys B (2015). Biomedical data sharing and reuse: attitudes and practices of clinical and scientific research staff. PLoS ONE.

[28] van Roode MY, Dos S Ribeiro C, Farag E (2024). Six dilemmas for stakeholders inherently affecting data sharing during a zoonotic (re-)emerging infectious disease outbreak response. BMC Infect Dis.

[29] Peñalvo JL, Mertens E, Ademović E (2021). Unravelling data for rapid evidence-based response to COVID-19: a summary of the unCoVer protocol. BMJ Open.

[30] Tacconelli E, Gorska A, Carrara E (2022). Challenges of data sharing in European Covid-19 projects: a learning opportunity for advancing pandemic preparedness and response. Lancet Reg Health Eur.

[31] Hallock H, Marshall SE, ’t Hoen PAC (2021). Federated networks for distributed analysis of health data. Front Public Health.

[32] Casaletto J, Bernier A, McDougall R, Cline MS (2023). Federated analysis for privacy-preserving data sharing: a technical and legal primer. Annu Rev Genomics Hum Genet.

[33] European Parliamentary Research Service (EPRS) (2023). European health data space: European parliament. https://www.europarl.europa.eu/RegData/etudes/ATAG/2023/754642/EPRS_ATA(2023)754642_EN.pdf.

[34] Lähteenmäki J, Vuorinen AL, Pajula J (2022). Integrating data from multiple Finnish biobanks and national health-care registers for retrospective studies: Practical experiences. Scand J Public Health.

[35] FINDATA social and health data permit authority pricing: FINDATA social and health data permit authority.

[36] Convention on Biological Diversity (United Nations) Nagoya protocol on access to genetic resources and the fair and equitable sharing of benefits arising from their utilization to the convention on biological diversity.

[37] World Health Organization (WHO) Bureau’s text of the WHO convention, agreement, or other international instrument on pandemic prevention, preparedness and response (WHO CA+) for the consideration of the Intergovernmental Negotiating Body at its fifth meeting: World Health Organization (WHO).

[38] (2023). DSI Scientific Network Using digital sequence information (DSI) to design an mRNA vaccine: COVID-19 case study: DSI Scientific Network.

[39] Eckhardt M, Hultquist JF, Kaake RM, Hüttenhain R, Krogan NJ (2020). A systems approach to infectious disease. Nat Rev Genet.

