# Supplementary material for: A Call for Action: Lessons Learned From a Pilot to Share a Complex, Linked COVID-19 Cohort Dataset for Open Science
Source: JMIR Public Health Surveill. 2025 Feb 11;11:e63996. doi: 10.2196/63996 (PMC11835595; doi:10.2196/63996)
Supplement: Multimedia Appendix 1 [file publichealth-v11-e63996-s001.docx]

**Supplementary Material**

*Timeline*


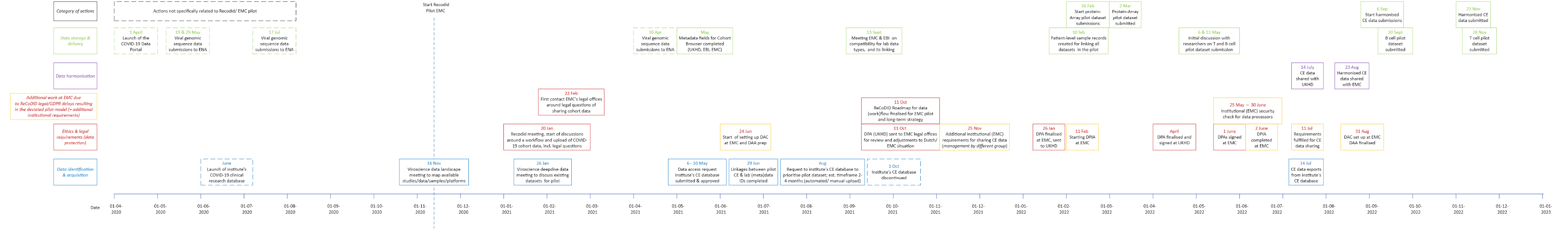


**First part of the analytical timeline is marked by continuous talks with Erasmus MC lab and clinical researchers, legal offices, the institutional clinical database team, and EBI**: The timeline is depicted at the bottom, with actions chronologically positioned and clustered according to the type of actions they related to. Actions related to the identification and acquisition of the data are depicted within blue boxes; actions related to ethics and legal requirements regarding data protection and privacy in red, with additional actions related to ethics and legal requirements performed at Erasmus MC due to GDPR delays (deviated pilot model) in yellow, while actions related to the data storage and delivery through a centralised approach are depicted in green boxes. Dashed boxes represent actions that were not specifically executed in the context of this pilot but had an influence on the timeliness of (other) actions in the pilot. DAC: data access committee; DAA: data access agreement; DPA: data processing agreement; DPIA: data protection impact assessment.


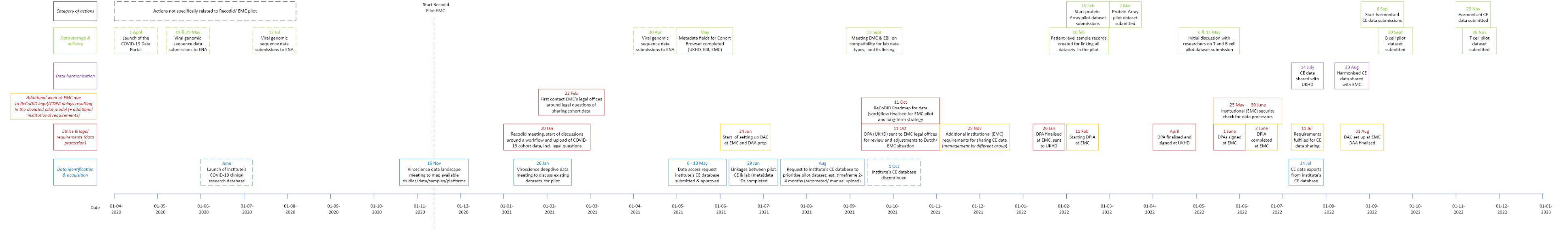


**Second part of the analytical timeline is marked by actions to adhere to legal requirements in collaboration with legal offices, data harmonisation of CE data in collaboration with UKHD, and finally data submission to in collaboration with the researchers and EBI**: The timeline is depicted at the bottom, with actions chronologically positioned and clustered according to the type of actions they related to. Actions related to the identification and acquisition of the data are depicted within blue boxes; actions related to ethics and legal requirements regarding data protection and privacy in red, with additional actions related to ethics and legal requirements performed at Erasmus MC due to GDPR delays (deviated pilot model) in yellow; actions related to (external) data harmonisation in purple boxes, while actions related to the data storage and delivery through a centralised approach are depicted in green boxes. Dashed boxes represent actions that were not specifically executed in the context of this pilot, yet had an influence on the timeliness of (other) actions in the pilot. DAC: data access committee; DAA: data access agreement; DPA: data processing agreement; DPIA: data protection impact assessment.
